# Circ_BBS9 as an early diagnostic biomarker for lung adenocarcinoma: direct interaction with IFIT3 in the modulation of tumor immune microenvironment

**DOI:** 10.3389/fimmu.2024.1344954

**Published:** 2024-07-19

**Authors:** Daijun Peng, Mingyu Liang, Lingyu Li, Haisheng Yang, Di Fang, Lingling Chen, Bing Guan

**Affiliations:** ^1^ Department of Pathology, Jinshan Branch of Shanghai Sixth People’s Hospital, Shanghai, China; ^2^ Department of Automation, Shanghai Jiao Tong University, Shanghai, China

**Keywords:** circular ribonucleic acids (circRNAs), lung adenocarcinoma (LUAD), circ_BBS9, ferroptosis, IFIT3, immune microenvironment

## Abstract

**Background:**

Introduction: Circular RNAs (circRNAs) have been identified as significant contributors to the development and advancement of cancer. The objective of this study was to examine the expression and clinical implications of circRNA circ_BBS9 in lung adenocarcinoma (LUAD), as well as its potential modes of action.

**Methods:**

The expression of Circ_BBS9 was examined in tissues and cell lines of LUAD through the utilization of microarray profiling, quantitative real-time polymerase chain reaction (qRT-PCR), and western blot analysis. In this study, we assessed the impact of circ_BBS9 on the proliferation of LUAD cells, as well as its influence on ferroptosis and tumor formation. To analyze these effects, we employed CCK-8 assays and ferroptosis assays. The identification of proteins that interact with Circ_BBS9 was achieved through the utilization of RNA pull-down and mass spectrometry techniques. A putative regulatory network comprising circ_BBS9, miR-7150, and IFIT3 was established using bioinformatics study. The investigation also encompassed the examination of the correlation between the expression of IFIT3 and the invasion of immune cells.

**Results:**

Circ_BBS9 was significantly downregulated in LUAD tissues and cell lines. Low circ_BBS9 expression correlated with poor prognosis. Functional experiments showed that circ_BBS9 overexpression inhibited LUAD cell proliferation and promoted ferroptosis *in vitro* and suppressed tumor growth *in vivo*. Mechanistically, circ_BBS9 was found to directly interact with IFIT3 and regulate its expression by acting as a sponge for miR-7150. Additionally, IFIT3 expression correlated positively with immune infiltration in LUAD.

**Conclusion:**

Circ_BBS9 has been identified as a tumor suppressor in lung adenocarcinoma (LUAD) and holds promise as a diagnostic biomarker. The potential mechanism of action involves the modulation of ferroptosis and the immunological microenvironment through direct interaction with IFIT3 and competitive binding to miR-7150. The aforementioned findings offer new perspectives on the pathophysiology of LUAD and highlight circ_BBS9 as a potentially valuable target for therapeutic interventions.

## Introduction

Lung cancer, being a prominent contributor to deaths due to cancer on a global scale (1), poses substantial challenges. Non-small cell lung cancer (NSCLC) comprises the majority, approximately 85%, of reported cases among the various histological types of lung cancer. Lung adenocarcinoma (LUAD), which is a specific subtype of non-small cell lung cancer (NSCLC), accounts for approximately 40% of all cases of lung cancer. The majority of LUAD cases arise from the glandular epithelium located in the peripheral regions of the lung (2). Despite significant advancements in contemporary technologies in the field of lung cancer treatment during the previous decade, there are still persistent challenges that hinder progress, as evidenced by the relatively low 5-year survival rate of approximately 10%-20% (2). In recent years, there have been notable developments in the field of medical treatments, specifically in targeted therapies like erlotinib, immunotherapy options such as pembrolizumab or nivolumab, and the utilization of combined surgical procedures (3, 4). However, the current limitations of conventional tumor markers, including their relatively lower specificity and sensitivity, contribute to the persistently low overall 5-year survival rate (OS) observed in late-stage LUAD patients (5, 6). Therefore, it is imperative to conduct comprehensive research on the tumorigenesis and progression mechanisms of LUAD in order to identify novel and more efficient diagnostic and therapeutic biomarkers that can improve the prognosis of patients with LUAD.

Circular RNAs (circRNAs) are a subclass of non-coding RNAs that are produced via back-splicing, an atypical splicing process (7). The 5’ and 3’ ends of linear mRNA are joined in this manner to create a circular structure. Circular RNAs are complex molecules that perform multiple functions, including post-transcriptional regulation and gene transcription regulation. Furthermore, they display a wide range of expression patterns that are specific to different tissues and developmental stages. They are associated with various normal and pathological conditions, particularly implicated in cancer pathogenesis. With the advancements in high-throughput RNA sequencing (RNA-seq) and bioinformatics, numerous functional circular RNAs have been discovered (8). Some functionally characterized circRNAs play critical roles in gene regulation through various mechanisms, such as acting as “sponges” for miRNAs, interacting with proteins, and regulating transcription and splicing. Circular RNAs (circRNAs) have been observed to engage in interactions with various RNA-binding proteins (RBPs), leading to the regulation of associated protein activity and the manifestation of a wide range of biological functions (9, 10).

In recent years, circRNAs have drawn substantial attention due to their prospective applications in cancer diagnosis, prevention, targeted therapy, and their role as dependable diagnostic and prognostic biomarkers. Numerous circRNAs manifest tumor-specific functionalities within cancers, playing a contributory role in modulating cancer progression and metastasis. Research suggests that the aberrant expression of circRNAs is intricately linked to various critical aspects, such as the activation of the PI3K/AKT signaling pathway, facilitation of cell cycle progression, promotion of metastasis, and modulation of anti-tumor immunity across diverse cancers, encompassing lung adenocarcinoma and others (11–14). These circular RNAs also actively contribute to the advancement and immune evasion strategies of various malignant tumors, including colorectal cancer, breast cancer, gastric cancer, hepatocellular carcinoma, among others (15–18). The dysregulated expression of circRNAs plays a pivotal role in the progression of cancer. The distinctive expression patterns of these circRNAs exhibit promising diagnostic potential and could emerge as viable targets for therapeutic interventions. Hence, a comprehensive comprehension of the biological functions and roles of circRNAs in distinct cancer types, along with their influence on signaling pathways, holds immense significance for early cancer detection and precision-based therapies.

Iron-dependent cell death, termed ferroptosis, represents a distinct form of programmed cell death that is iron-reliant and exhibits notable disparities from apoptosis and autophagy (19). It hinges on iron-mediated oxidative injury, heightened accumulation of iron, generation of free radicals, and the supply and buildup of lipid peroxides in fatty acids (20). Various studies have implicated connections between ferroptosis and diverse conditions, encompassing cancer, neurological disorders, and infections (19, 21, 22). Its defining features encompass the accrual of reactive oxygen species (ROS) and the depletion of glutathione. In contrast to apoptosis, necrosis, and autophagy, ferroptosis presents marked distinctions in cellular structure and function. Its mechanism relies on iron-facilitated lipid peroxidation and is intricately governed by numerous cellular metabolic and signaling pathways (23). The principal mechanisms underlying ferroptosis revolve around the catalytic action of ferrous iron or lipoxygenases, leading to the peroxidation of highly abundant unsaturated fatty acids situated on the cellular membrane, ultimately instigating cell demise. Distinctive features characterizing ferroptosis encompass escalated lipid ROS levels, intracellular accumulation of ferrous iron ions, buildup of lipid peroxides, and the decreased expression of factors that inhibit ferroptosis, such as glutathione peroxidase 4 (GPX4) and ferritin heavy chain 1 (FTH1) (23). Downregulation of GPX4 expression stands as a pivotal factor in impeding ferroptosis, functioning by scavenging lipid ROS and thwarting GPX4-mediated cell demise. This factor exhibits a close association with tumor progression (24, 25). FTH1, an integral constituent of ferritin crucial for preserving intracellular iron equilibrium, serves to prevent adverse consequences arising from iron overload (26, 27). Consequently, inhibiting FTH1 might potentially contribute to the induction of ferroptosis.

It’s noteworthy that the regulation of circRNAs is also linked to ferroptosis in the context of cancer progression, potentially opening new avenues for future cancer therapies (28). Although circRNAs play a critical regulatory role in tumor cell metabolism, their specific involvement in the atypical metabolism associated with tumor-induced ferroptosis remains incompletely understood. Advancing research in this domain holds the promise of unveiling the potential contribution of circRNAs to cancer therapy.

For a long time, chemotherapy, radiation therapy, and surgery have been the mainstays of cancer treatment, achieving significant progress. However, these traditional treatment methods exhibit notable limitations in patients with advanced or metastatic malignancies and often come with severe side effects. To confront these challenges, immunotherapy stands out as an immensely promising approach that is gradually gaining traction. It works by reinvigorating immune surveillance and promoting the immune system’s clearance of tumors (29). The tumor microenvironment (TME) is defined as a complex, diverse multicellular environment crucial for tumor development, typically composed of various immune cells, including T and B lymphocytes, tumor-associated macrophages (TAMs), dendritic cells (DCs), natural killer cells (NK cells), neutrophils, and myeloid-derived suppressor cells (MDSCs) (29). Tumor-infiltrating T cells play a crucial role in molding a favorable TME. Nevertheless, regulatory T cells (Tregs) exert immune-suppressive functions by releasing factors like IL-10 and transforming growth factor-beta (TGF-β). These actions assist cancer cells in evading immune defenses (30). Within lung cancer, tumor cells additionally express immune-inhibitory factors such as IL-10 and TGF-β, thereby fostering the recruitment of regulatory T cells (Tregs) and MDSCs (31, 32). The immune microenvironment significantly influences the initiation, infiltration, and metastasis of tumors, exerting a pivotal impact on cancer diagnosis, prevention, and prognosis (33, 34).

With the rapid development of nanomedicine, various functional dendritic macromolecules and dendritic macromolecule-based nanohybrids have been explored in the treatment and diagnosis of cancer. Recently, they have shown great promise in cancer immunotherapy, providing more opportunities for efficient cancer immunotherapy (35). Studies have investigated the intrinsic immune-modulating effects of tumor-targeted nano adjuvants and their ability to simultaneously trigger the release of tumor antigens, thereby reversing immune suppression and achieving potent antitumor immunity, with significant application potential in breast cancer treatment (36). The emergence of these novel approaches in cancer therapeutics, immunotherapy presents a beacon of hope for patients grappling with lung adenocarcinoma, potentially extending their survival rates. Despite these significant advancements, cancer still finds ways to counteract any treatment strategy through dynamic evolution and developing mechanisms of drug resistance. The intricacies inherent in lung adenocarcinoma have led to a constrained scope of research findings, resulting in a dearth of standardized tools capable of effectively guiding clinical decisions in this complex domain. It is essential to further study and develop prognostic biomarkers for lung adenocarcinoma. Therefore, gaining a deeper understanding of the interactions between tumor cells and host immune cells within the tumor microenvironment will help us better comprehend how tumors evade attacks from the immune system, thereby driving the development of precision medicine and individualized combined immunotherapy.

Bioinformatics methodologies have notably contributed to our research endeavors. Through the exploration of immune-related genes, we’ve achieved a more profound comprehension of the intricate relationship and interaction pathways linking LUAD with the immune microenvironment. This holds promise for inspiring early diagnosis, improving prognosis, and developing new therapeutic targets (37). We performed microarray analyses on expression profile datasets pertaining to lung adenocarcinoma and circular RNA expressions. Via meticulous experimental validation, we successfully identified differentially expressed circular RNAs and delineated their potential roles in the pathogenesis of LUAD. In this study, we amalgamated diverse lung adenocarcinoma sample datasets at multiple levels, incorporating clinical data and database analyses. Leveraging several well-established bioinformatics analysis tools, we investigated the correlation between the expressions of circ_BBS9 and IFIT3 and their implications on prognosis. We identified that overexpression of circ_BBS9 inhibits lung adenocarcinoma cell proliferation and promotes ferroptosis of lung adenocarcinoma cells. Additionally, the protein IFIT3, which directly interacts with circ_BBS9, is involved in immune infiltration and participates in the formation of the immune microenvironment. We also established a potential transcriptional network involving “circ_BBS9”-”hsa-miR-7150”-”IFIT3,” which might be involved in the pathogenesis of LUAD. Ultimately, our research uncovered the promising potential of circ_BBS9 as a groundbreaking biomarker for early diagnosis and treatment strategies. Furthermore, our identification of the direct interaction between circ_BBS9 and IFIT3 sheds light on their collective role in shaping the immune microenvironment within LUAD. These discoveries unveil novel molecular mechanisms and offer potential therapeutic targets, presenting extensive opportunities in advancing the diagnosis and treatment of LUAD.

## Materials and methods

### Clinical tissue collection

All validation samples were collected with the consent of the patients and obtained ethical approval from the Jinshan Branch of the Shanghai Sixth People’s Hospital, China. All patients were diagnosed with LUAD based on their histological and pathological characteristics. Clinical samples included tumor tissues and adjacent non-tumor lung tissues. None of the patients had received any preoperative chemotherapy or radiation therapy. Excised tissues were stored at -80°C for long-term preservation.

### Expression analysis

The data were sourced from The Cancer Genome Atlas (TCGA: https://cancergenome.nih.gov) and the Gene Expression Omnibus (GEO: https://www.ncbi.nlm.nih.gov/geo/) datasets (GSE101684, GSE112214, GSE101586, GSE116959, and GSE72094). Protein expression analysis was conducted using the Human Protein Atlas (https://www.proteinatlas.org) to assess the expression of circ_BBS9 and IFIT3 proteins in lung adenocarcinoma patients.

A comprehensive analysis of circ_BBS9 and IFIT3 expression necessitated the utilization of various resources, including Tumor Immune Estimation Resource (TIMER: https://cistrome.shinyapps.io/timer/) (38), Gene Expression Profiling Interactive Analysis (GEPIA: http://gepia.cancer-pku.cn/) (39), and UALCAN (http://ualcan.path.uab.edu/home) (40). The gene expression profile interaction analysis of BBS9 and IFIT3 in lung adenocarcinoma was performed using GEPIA (http://gepia.cancer-pku.cn/).

### Analysis of differentially expressed genes

Different Expressed Genes (DEGs) data from 3 GEO databases (GSE101684, GSE112214, GSE101586) of LUAD was extracted and processed using Python package “pandas”,”scipy” and some other essential packages. Statistical analyses were also calculated with python (| log_2(FC)| > 1, adj.P<0.05). Finally, the results were visualized with the ChiPlot tools.

### Survival and prognostic analysis

The survival package and survminer package were employed to assess the correlation between expression levels and the survival rate associated with circ_BBS9 across various clinical features within the GEO dataset (GSE72094). The ROC package was utilized to construct the Receiver Operating Characteristic (ROC) curve for diagnostic purposes.

### Enrichment analyses

To uncover potential mechanisms, we conducted Gene Ontology (GO) and Kyoto Encyclopedia of Genes and Genomes (KEGG) analyses with OmicShare tools (https://www.omicshare.com/tools). Genes associated with IFIT3 were collected using STRING (https://cn.string-db.org/) and GeneMania (https://genemania.org/).

### Gene set enrichment analysis

We conducted Gene Set Enrichment Analysis (GSEA) employing OmicShare tools (https://www.omicshare.com/tools). Enrichment map analysis was subsequently applied to interpret the GSEA results. Significance was determined by a nominal p-value < 0.05, and a false discovery rate (FDR) q-value<0.25.

### RNA extraction and real-time PCR

Total RNA from the cells was extracted using TRIzol™ (ThermoFisher, USA), followed by reverse transcription using the first-strand cDNA synthesis kit (TaKaRa, Japan). Subsequently, real-time polymerase chain reaction (RT-PCR) was conducted.

The primers are as follows:

has_circ_0049271 forward: 5`-AACTTCGCTGAGCAGATTGG-3`,

reverse: 5`-TAAGCAACACCACCACCTCT-3`;

has_circ_0004789 forward: 5`-CCATCAACCGCCTCAAAGAC-3`,

reverse: 5`-TTGCCCAGATCCATCAACCA-3`;

has_circ_0003162 forward: 5`-CTGTCTCAGGAACCTTGGG-3`,

reverse: 5`-CCACCAATCACGGGCTTTAA-3`;

has_circ_0061817 forward: 5`-CCTGTCCTCCTAAACCTCCA-3`,

reverse: 5`-TCTCGCTGACCAAGAACTGA-3`;

has_circ_0015278 forward: 5`-TACAACCCCAGAACCAACCA-3`,

reverse: 5`-AGAACACTGACCCCAACTCC-3`.

GAPDH was used as an internal control.

### Cell culture

Lung normal epithelial cells (BEAS-2B) and lung cancer cell lines (A549, H1299) were obtained from ATCC (Manassas, USA). These cell lines were cultured in RPMI-1640 medium (Gibco, USA) supplemented with 10% fetal bovine serum (FBS, Biological Industries, Israel) and 1% penicillin-streptomycin solution (Solarbio, China). The cells were maintained in a humidified incubator at 37°C with 5% CO2.

### Cell transfection

#### H1299

In this experiment, we used Lipofectamine™ 2000 transfection reagent to transfect hsa-circ-0003162-1 and NC into H1299 cells, as shown in **Table 1**. One day prior to transfection, cells were seeded into a 6-well plate at a density of 5 x 10^5 cells per well. After 24 hours, the cell confluency was observed to reach 80% to 90%, at which point hsa-circ-0003162-1 was transfected.

**Table 1:** The sequences of hsa-circ-0003162 and negative control.

	Sequence (5’to 3’)
hsa-circ-0003162-1	GGUGUAAAAGUGUGAAAGATT UCUUUCACACUUUUACACCTT
Negative control	UUCUCCGAACGUGUCACGUTT ACGUGACACGUUCGGAGAATT

Medium Replacement: The original medium in each well was discarded and replaced with fresh medium. Transfection reagent and RNA were diluted with OPTI-MEM and incubated at room temperature for 5 minutes. The amount of transfection reagent and OPTI-MEM added per well is shown in **Table 2**. The 50 μL diluted interference fragments and 50 μL diluted transfection reagent were mixed well and incubated at room temperature for 15 minutes. The 100 μL incubated mixture was then added to the cell samples. After 6 hours, the medium was replaced with fresh medium.

**Table 2:** Transfection reagent and OPTI-MEM added per well in H1299 cells.

	Reagent Dosage	OPTI-MEM
Lipofectamine™ 2000	20 μl	180 μl
hsa-circ-0003162-1	5 μl	45 μl
NC	5 μl	45 ul

#### A549

Using Lipofectamine™ 2000 transfection reagent, the circ-0003162 overexpression plasmid and empty vector were transfected into A549 cells. One day prior to transfection, cells were seeded into a 6-well plate at a density of 3 x 10^5 cells per well. After 24 hours, the cell confluency was observed to reach 80% to 90%, at which point the circ-0003162 plasmid was transfected.

Medium Replacement: The original medium in each well was discarded and replaced with fresh medium. The transfection reagent and plasmid were diluted with OPTI-MEM and incubated at room temperature for 5 minutes. The amount of transfection reagent and OPTI-MEM added per well is shown in **Table 3**. The 50 μL diluted plasmid and 50 μL diluted transfection reagent were mixed well and incubated at room temperature for 20 minutes. The 100 μL incubated mixture was then added to the cell samples. After 6 hours, the medium was replaced with fresh medium.

**Table 3:** Transfection reagent and OPTI-MEM added per well in A549 cells.

	Reagent Dosage	OPTI-MEM
Lipofectamine™ 2000	10μl	90μl
pD circ–0003162	2ug	Up to 50μl
pD circ	2ug	Up to 50ul

### Cell counting kit 8 assay

To assess cell viability, a Cell Counting Kit-8 (CCK-8) assay kit was employed following the manufacturer’s instructions. Briefly, cells were seeded in 96-well plates at a density of 1000 cells per well, in culture medium containing 10% fetal bovine serum and penicillin-streptomycin (5000 U/mL), and were maintained at 37°C in a humidified atmosphere with 5% CO2. After 24 hours of incubation, 10 μL of CCK-8 reagent was added to each well of the 96-well plates and incubated for 2 hours at 37°C in a humidified atmosphere with 5% CO2. The absorbance of each well was measured at 450 nm using a microplate reader.

### Immunofluorescence assays

Cells were seeded onto 24-well plates. After fixation (4% paraformaldehyde, 15 minutes at room temperature) and blocking (3% BSA, 30 minutes at room temperature), the primary antibody (1:250, Abcam, derived from rabbit) was incubated at 4°C overnight. Subsequently, the corresponding fluorescent secondary antibody (1:500, Abcam, derived from goat) was incubated for 1 hour at room temperature. Anti-fade 4`,6-diamidino-2-phenylindole (DAPI) was employed for cell nuclei labeling. Images were captured using a fluorescence microscope.

### Measurement of ROS

Dihydroethidium (Beyotime, #S0063) was employed as a molecular probe for detecting ROS in red fluorescence. FerroOrange (DOJINDO, #F374) was quantified using a flow cytometer or visualized under a fluorescence microscope.

### Measurement of MDA levels

To measure MDA levels, a lipid peroxidation assay kit was employed according to the manufacturer’s instructions. Briefly, 1 × 10^6 cells were collected in 300 μL of MDA lysis buffer containing 3 μL of butylated hydroxytoluene (BHT, 100×, to reduce interfering lipid oxidation), and the samples were homogenized on ice. After centrifugation at 13,000 g for 10 minutes, the insoluble material was removed. Subsequently, 600 μ L of thiobarbituric acid (TBA) solution, which reacts with other compounds in the samples to produce colored products, was added to each experimental sample or vial containing a standard sample. The MDA-TBA adducts were allowed to form by incubating for 60 minutes at 95°C. After cooling to 25°C in an ice bath, 200 μ L of each reaction mixture was pipetted into a 96-well plate for colorimetric assays, and the absorbance was measured at 532 nm.

### Fe^2+^ content measurement

Following cell treatment based on the grouping, the cells were washed twice with FBS-free DMEM. Subsequently, a working solution of Ferro Orange (1 μmol/L; excitation wavelength: 540 nm, emission wavelength: 580 nm; Dojindo, Kumamoto, Japan) was prepared using FBS-free DMEM as per the manufacturer’s instructions. The solution was then incubated at 37°C in a 5% CO2 incubator for 30 minutes and finally photographed using a multifunctional microplate detection system (CYTATION5, BIOTEK, USA).

### Protein extraction and western blot

Cells were collected, washed with ice-cold phosphate-buffered saline (PBS), and lysed for 30 minutes in RIPA buffer containing 50mM Tris/HCl (pH 7.5), 150mM NaCl, 1% NP40, 1% Triton X-100, 2.5mM sodium pyrophosphate, 1mM β-glycerophosphate, 1mM EDTA, 1mM Na3VO4, and 1μg/mL leupeptin. Cell lysates were then centrifuged at 14,000g for 10 minutes at 4°C, and the protein concentration was measured using the BCA Protein Assay kit (Pierce, Rockford, IL). Aliquots of lysates (twenty micrograms of protein) were boiled with sample loading buffer (Beyotime; P0015) for 5 minutes and resolved by sodium dodecyl sulfate-polyacrylamide gel electrophoresis (SDS-PAGE). After electrophoresis, proteins were electrophoretically transferred onto a polyvinylidene difluoride (PVDF; Roche) membrane using a Semi-Dry Electroblotter (Bio-Rad). Following transfer, the membrane was blocked for 2 hours at room temperature in phosphate-buffered saline (PBS) containing 5% (w/v) nonfat milk and 0.1% (v/v) Tween-20. The membranes were then incubated with primary antibodies against human GPX4 (1:5000, Abcam, USA), GAPDH (1:500, Proteintech, China), FTH1 (1:1000, Abcam, USA)and IFIT3 (1:2000, CST, USA)at 4°C overnight, followed by a 1-hour incubation at room temperature with horseradish peroxidase (HRP)-linked anti-rabbit secondary antibody (Proteintech, SA00001–2; 1:50000) or anti-mouse secondary antibody (Proteintech, SA00001–1; 1:100000). After four washes with PBS containing 0.1% (v/v) Tween-20, immunoreactive bands were visualized using Chemistar™ High-sig ECL Western Blotting Substrate (Tanon; 180–501).

### Pull-down assay

A biotin-labeled oligonucleotide probe specific to circ_BBS9 was synthesized commercially (RiboBio, China). In brief, the biotin-labeled oligonucleotide probes were incubated with BeyoMag™ streptavidin magnetic beads (Beyotime; P2151) for 60 minutes at room temperature. Once bound to the streptavidin magnetic beads, the probe-beads were incubated with whole cell lysates overnight at 4°C. Following three washes with ice-cold PBS, miRNAs or proteins that were pulled down by the probe-coated beads were collected.

### RNA immunoprecipitation assay

To perform the RNA immunoprecipitation (RIP) assay, cells were harvested and resuspended in 1 mL of lysis buffer containing a protease inhibitor cocktail and RNase inhibitor. After centrifugation at 13,000 rpm for 10 minutes at 4°C, the supernatant was incubated with 30-40 μL of Protein A-Sepharose beads (Genescript) and 2 μg of primary antibodies (Proteintech, derived from rabbit) for 4 hours at 4°C. Subsequently, the beads were washed with ice-cold 1×PBS. Following this, the beads were incubated with Proteinase K (Sigma) using Trizol reagent (Invitrogen Life Technologies), and the purified RNA was subjected to qRT-PCR analysis.

### TF-miRNA-mRNA regulatory network

Transcription factors (TFs) targeting IFIT3 were predicted based on CHEA (https://maayanlab.cloud/Harmonizome/) and GRNdb (http://www.grndb.com/). miRNAs targeting IFIT3 were predicted based on three different databases: miRWalk (http://mirwalk.umm.uni-heidelberg.de/), TargetScan (https://www.targetscan.org/vert_80/), and mirDIP (http://ophid.utoronto.ca/mirDIP/). StarBase V2.0 (https://starbase.sysu.edu.cn/index.php) was used to predict miRNA expression levels, prognostic value, and interaction sites. The final results were visualized by initially employing the venn3 function in in matplotlib_venn in python for creating a Venn diagram. Subsequently, based on the diagram results, graphical adjustments were made using the ‘pyplot’ module from Matplotlib to generate a comprehensive visualization.”

### Immune infiltration analysis

The relationship between immune infiltration and IFIT3 was assessed using TIMER 2.0 (38) and TISIDB (41). In brief, TIMER 2.0 was employed to elucidate the association between GPER and tumor-infiltrating immune cells (TIICs). TISIDB elucidated the relationship between tumor-infiltrating lymphocyte (TIL) abundance and IFIT3 expression.

### Statistical analysis of data

In this study, different statistical tests were employed for data analysis, depending on the data characteristics and comparison requirements:

(1). For the comparison of expression levels between two groups, an unpaired t-test was employed.
(2). When dealing with more than two groups, an initial Analysis of Variance (ANOVA) test was conducted to assess whether significant differences existed among all groups. Subsequently, based on the inter-group variations obtained through Tukey’s multiple comparisons test, a secondary unpaired t-test was performed to ascertain significant differences between two groups, yielding the final statistical conclusions depicted in the figures.
(3). For survival curve statistical analysis, the Log-Rank (Mantel-Cox) method was used to compare two curves and evaluate statistical differences.

In this paper, significance levels (*, **, ***) are used to indicate p-values of less than 0.05, 0.01, and 0.001, respectively. Smaller p-values signify greater statistical significance in the differences between the compared data.

## Results

### Characteristics of differentially expressed CircRNAs in LUAD

**Figure 1:**
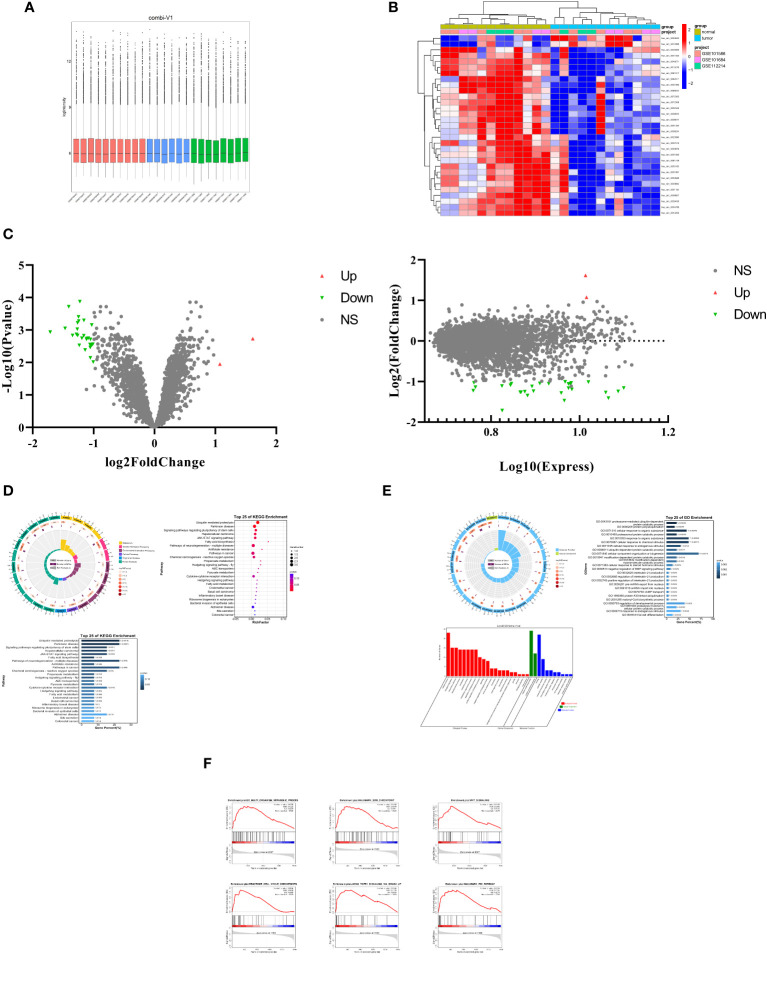
Characteristics of differentially expressed CircRNAs in LUAD. **(A)** The lung cancer expression microarray dataset from the GEO Database. **(B)** A cluster heap map presented the significantly dys regulated circRNAs in human LUAD tissues relative to adjacent normal tissues. The red and blue strips represent high and low expression, respectively. **(C)** Scatter plot and Volcano plot of differentially expressed circRNAs in LUAD and adjacent normal tissues. **(D)** Differential expression of circRNAs in KEGG pathway analysis results. **(E)** Differential expression of circRNAs in GO analysis results Results. **(F)** GSEA (Gene Set Enrichment Analysis) of differentially expressed circRNAs.

To investigate the role of circRNAs in the progression of lung adenocarcinoma, we conducted a merged analysis using lung adenocarcinoma expression microarray datasets from the GEO database (GSE101684, GSE11221, GSE101586) (**Figure 1A**). We employed a heatmap (**Figure 1B**) and a volcano plot (**Figure 1C**) to screen for differentially expressed circRNAs. In this process, a total of 31 differentially expressed circRNAs were identified, with the noteworthy observation that these circRNAs primarily exhibited a downregulation trend. Subsequently, we excluded two upregulated circRNAs and focused on the remaining 29 differentially expressed downregulated circRNAs for further investigation.

Through the study of these differentially expressed circRNAs, we conducted Kyoto Encyclopedia of Genes and Genomes (KEGG) analysis (**Figure 1D**). The results revealed that the downregulated circRNAs play important roles in ubiquitin-mediated proteolysis, cytokine-cytokine receptor interaction, metabolism, and the regulation of various signaling pathways. Furthermore, Gene Ontology (GO) term analysis (**Figure 1E**) also disclosed that these circRNAs are associated with biological processes (BP) related to cell metabolism, bioregulation, and stimulus response, cellular components (CC) involving cellular anatomical entities and protein-containing complexes, and molecular functions encompassing binding and catalytic activity.

Further research included Gene Set Enrichment Analysis (GSEA). This analysis (**Figure 1F**) demonstrated significant enrichment of these circRNAs in cellular metabolism, cell cycle, proliferation, invasion, antigen response, inflammation, and various signaling pathways. These findings underscore the crucial role of these downregulated circRNAs in the progression of lung adenocarcinoma, potentially affecting cell functions and disease mechanisms through multiple pathways.

### Expression and survival of circ_BBS9 in LUAD tissues

**Figure 2:**
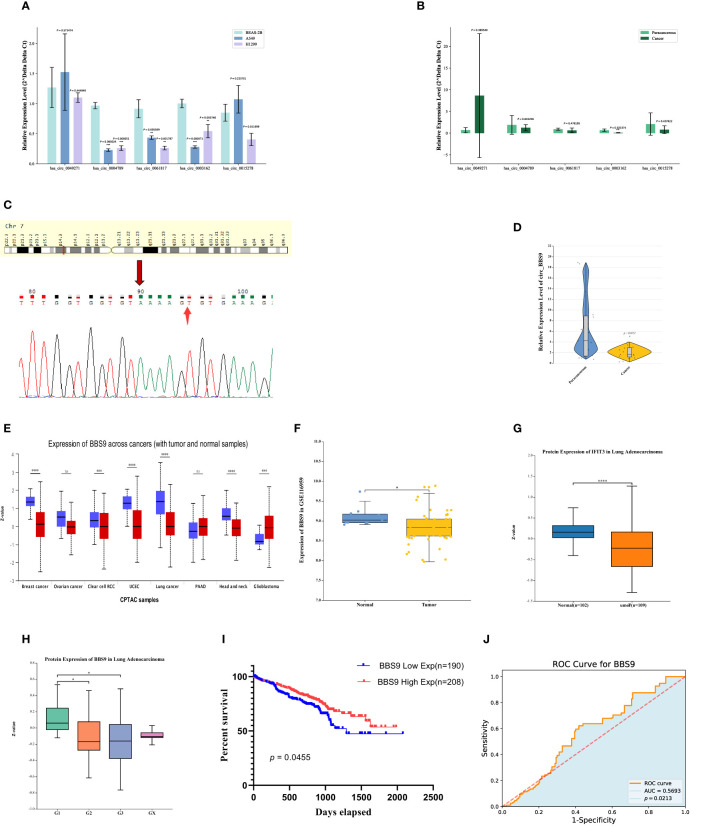
Expression and survival of Circ BBS9 in LUAD Tissues. **(A)** Expression of differentially downregulated circRNAs in A549, H1299, and (BEAS-2B) determined by qRT-PCR assays. **(B)** Expression of differentially downregulated circRNAs in clinical samples determined by qRT-PCR assays. **(C)** sequencing of PCR products revealing the circularization site of circRNA as indicated (Arrows) in the figure above. **(D)** Expression of circ BBS9 in 15 clinical samples as obtained by qRT PCR assays. **(E)** BBS9 expression in different cancer types analyzed by UALCAN. **(F)** In the GEO database, BBS9 exhibits lower mRNA expression levels in LUAD compared to normal tissues (*p* <0.05). **(G)** In the HAP database, the protein expression level of BBS9 is lower in LUAD compared to normal tissues (*p* < 0.001). **(H)** The expression of BBS9 in LUAD is correlated with Tumor Grade (*p* < 0.05). **(I)** Survival curves of overall survival in GSE72094 LUAD patients. **(J)** The predictive ability of variable BBS9 in LUAD.

For an in-depth investigation, we selected five circRNAs (has_circ_0049271, has_circ_0004789, has_circ_0003162, has_circ_0061817, has_circ_0015278) with the greatest downregulation and that had not been previously reported in the field of lung adenocarcinoma for further study. We employed qRT-PCR to assess the expression levels of these five downregulated circRNAs in two human lung adenocarcinoma cell lines (A549 and H1299) and one human normal lung epithelial cell line (BEAS-2B) (**Figure 2A**). The results indicated that three circRNAs (has_circ_0004789, has_circ_0061817, has_circ_0003162) exhibited low expression in lung adenocarcinoma cell lines.

To further validate these results, we collected clinical samples from 15 patients diagnosed with lung adenocarcinoma, comprising both cancer tissues and adjacent normal tissues. We used qRT-PCR to measure the mRNA levels of these three circRNAs (has_circ_0004789, has_circ_0061817, has_circ_0003162) in the cancer tissues and adjacent normal tissues of three patients (**Figure 2B**). The results indicated that has_circ_0003162 had significantly lower expression in cancer tissues compared to adjacent normal tissues. Therefore, we selected has_circ_0003162 as the subject for further investigation. These series of experimental findings underscore the potential importance of has_circ_0003162 in lung adenocarcinoma and provide a foundation for further mechanistic research.

Has_circ_0003162, derived from the BBS9 gene on chromosome 7, was unambiguously identified as our screened circ_BBS9 through divergent primer amplification and Sanger sequencing (**Figure 2C**). To validate our findings, we verified the expression of circ_BBS9 in cancer tissues and adjacent tissues of 12 additional lung adenocarcinoma patients (**Figure 2D**). The results showed a significant reduction in the expression level of circ_BBS9 in lung adenocarcinoma tissues.

To further explore the relationship between circ_BBS9 and LUAD, we used UALCAN to analyze the expression of BBS9 in various tumors (**Figure 2E**). We compared the expression levels of BBS9 in tumor tissues and adjacent normal tissues, revealing differential expression of circ_BBS9 in various tumor tissues. Using data from the GEO database (GSE116959), we assessed the mRNA expression of BBS9 in LUAD tissues and non-tumor tissues (**Figure 2F**) and validated the protein-level expression of BBS9 in LUAD through the Human Protein Atlas (HPA) (**Figure 2G**). We found a consistent trend of downregulated BBS9 expression in LUAD, regardless of the different databases or methods used for analysis. Furthermore, in different tumor grades, the expression of BBS9 was significantly lower in G2 and G3 compared to G1 (**Figure 2H**), suggesting a correlation between BBS9 and tumor grading.

Subsequently, we analyzed the prognosis of 398 lung adenocarcinoma patients from the GEO database who expressed the BBS9 gene (**Figure 2I**). The results indicated that patients with high circ_BBS9 expression had a better survival prognosis. Through receiver operating characteristic (ROC) curve analysis (**Figure 2J**), we confirmed the diagnostic value of circ_BBS9 in LUAD, with an area under the curve (AUC) of 0.5693, suggesting a strong correlation between circ_BBS9 and the diagnosis of LUAD. In summary, circ_BBS9 may serve as a potential biomarker with significant clinical implications for the diagnosis and prognosis of LUAD.

### Impact of circ_BBS9 on the proliferation of A549 and H1299 LUAD cells

**Figure 3:**
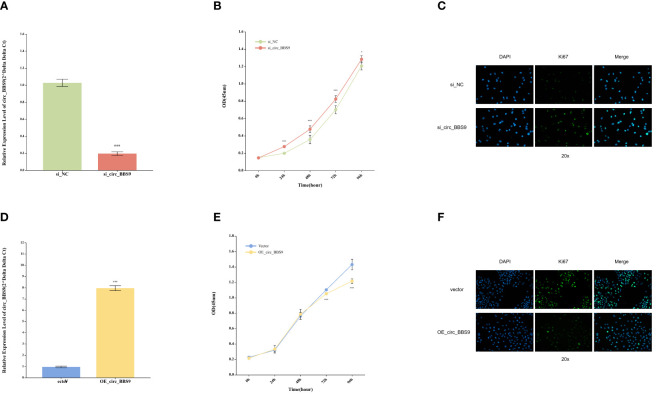
Impact of Circ_BBS9 on the Proliferation of A549 and H1299 LUAD Cells. **(A)** Comparison of circ_BBS9 expression levels between the negative control (NC) group and the si_circ_BBS9 group in H1299 cells using qRT-PCR. **(B)** Comparison of cell viability between the negative control (NC) group and si_circ_BBS9 group in H1299 cells using a CCK-8 assay. **(C)** Immunofluorescence detection of Ki67 cell proliferation in the negative control (NC) group and si_circ_BBS9 group in H1299 cells. **(D)** Comparison of circ_BBS9 expression levels between the Vector group and OE_circ _BBS9 group in A549 cells using qRT-PCR. **(E)** Comparison of cell viability between the Vector group and OE_circ_BBS9 group in A549 cells using CCK-8 assay. **(F)** Assessment of Ki67 cell proliferation in A549 cells for the Vector group and OE_circ_BBS9 group using immunofluorescence.

In order to assess the functional significance of circ_BBS9, we conducted *in vitro* experiments using A549 and H1299 LUAD cells. Employing siRNA interference techniques, we successfully reduced the expression levels of circ_BBS9 in H1299 cells (**Figure 3A**). Cellular viability was examined through CCK-8 assays (**Figure 3B**), revealing a significant increase in cell proliferation in the si_circ_BBS9 group. Furthermore, immunofluorescence analysis of H1299 cells was performed to detect Ki67-positive cell nuclei (displayed as green fluorescence) (**Figure 3C**), and the results indicated a noteworthy elevation in the percentage of Ki67-positive cell nuclei in the si_circ_BBS9 group. This suggests that circ_BBS9 promotes the proliferation of LUAD cells.

Simultaneously, we employed the overexpression technique (OE-RNA) to overexpress circ_BBS9 in A549 cells, successfully elevating the expression levels of circ_BBS9 (**Figure 3D**). In the CCK-8 assay, the OE_circ_BBS9 group exhibited a noticeable decrease in cell proliferation (**Figure 3E**). Immunofluorescence analysis revealed a significant reduction in the percentage of Ki67-positive cell nuclei in the OE_circ_BBS9 group compared to the control group (**Figure 3F**). These findings indicate that OE_circ_BBS9 inhibits the proliferation of LUAD cells.

These results underscore the crucial regulatory role of circ_BBS9 in LUAD cells. Its downregulation enhances cell proliferation, while its overexpression suppresses cell proliferation, emphasizing the potential involvement of circ_BBS9 in the pathophysiological processes of LUAD.

### The effect of circ_BBS9 on ferroptosis in A549 and H1299 LUAD cells

**Figure 4:**
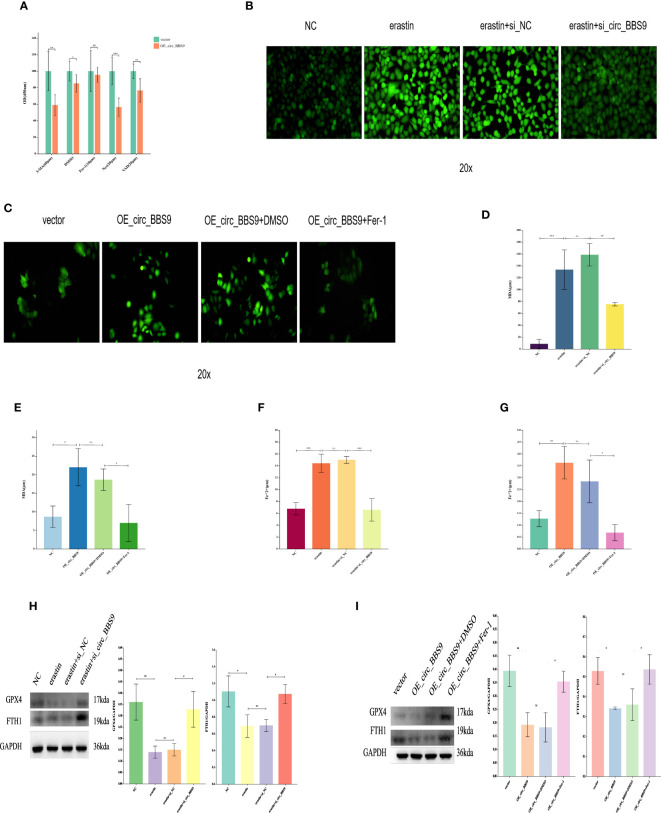
The Effect of Circ_BBS9 on Ferroptosis in A549 and H1299 LUAD Cells. **(A)** CCK-8 assay to determine the cell death mechanism in lung adenocarcinoma cells. **(B)** Detection of ROS in H1299 cells using a fluorescence probe. **(C)** Detection of ROS in A549 cells using a fluorescence probe. **(D)** Measurement of MDA content in H1299 cells using a colorimetric assay. **(E)** Measurement of MDA content in A549 cells using a colorimetric assay. **(F)** Measurement of iron ion concentration in H1299 cells using a colorimetric assay. **(G)** Measurement of iron ion concentration in A549 cells using a colorimetric assay. **(H)** Detection of ferroptosis inhibitor GPX4 and FTH1 expression in H1299 cells using Western Blot method. **(I)** Detection of ferroptosis inhibitor GPX4 and FTH1 expression in A549 cells using Western Blot method.

To further assess the impact of circ_BBS9 on LUAD progression, a series of experiments were conducted. Initially, we investigated whether known cell death inhibitors would affect the mode of cell death in OE_circ_BBS9 LUAD cells. Various cell death inhibitors, including the Ferroptosis inhibitor ferrostatin-1, apoptosis inhibitor Z-VAD-fmk, necroptosis inhibitor necrostatin-1 (Nec-1), and autophagy inhibitor 3-MA, were employed to determine the mode of cell death in OE_circ_BBS9 LUAD cells through CCK-8 assays (**Figure 4A**). We transfected the circ_BBS9 overexpression plasmid into A549 cells and observed that DMSO, VAD, Nec, and 3-MA significantly inhibited cell proliferation. However, the Ferrostatin-1 group did not impact the proliferation of LUAD cells. Thus, we ruled out other known modes of cell death and proceeded with investigating ferroptosis.

The process of ferroptosis is often accompanied by an increase in ROS. Therefore, we employed fluorescence probe-based detection to assess ROS expression and observe the occurrence of ferroptosis. The addition of the ferroptosis inducer erastin in H1299 cells led to an elevation in ROS. However, the introduction of erastin in the si_circ_BBS9 group resulted in reduced ROS expression (**Figure 4B**), indicating that si_circ_BBS9 inhibits ferroptosis in LUAD cells. In A549 cells, OE_circ_BBS9 caused an increase in ROS levels. When we added the ferroptosis inhibitor Fer-1 to the OE_circ_BBS9 group, it lowered the ROS expression induced by overexpression, thereby suppressing ferroptosis (**Figure 4C**). These results demonstrate that OE_circ_BBS9 promotes ferroptosis in LUAD cells.

Malondialdehyde (MDA) is a natural product of lipid oxidation in biological organisms. The measurement of MDA is widely used as an indicator of the extent of lipid oxidation, and it can be employed to assess the degree of lipid peroxidation. We observed an increase in MDA concentration after introducing the erastin in H1299 cells, while adding the erastin inducer to the si_circ_BBS9 group led to a decrease in MDA concentration (**Figure 4D**). This indicates that si_circ_BBS9 inhibits ferroptosis in LUAD cells. In A549 cells overexpressing OE_circ_BBS9, MDA concentration increased, but when we added the Fer-1 to the OE_circ_BBS9 group, MDA concentration decreased (**Figure 4E**). These results demonstrate that OE_circ_BBS9 promotes ferroptosis in LUAD cells.

Iron ions form complexes with proteins, and in acidic environments, iron dissociates from the complexes and is then reduced to ferrous iron. It eventually forms a purple-red compound with ferrozine, which can be quantified through colorimetry in the wavelength range of 540-580 nm. This method is used to measure iron ion concentration. In H1299 cells, the introduction of the erastin resulted in an increase in ferrous iron ion concentration, whereas adding the erastin inducer to the si_circ_BBS9 group led to a decrease in ferrous iron ion concentration (**Figure 4F**). This indicates that si_circ_BBS9 inhibits ferroptosis in LUAD cells. In A549 cells overexpressing OE_circ_BBS9, ferrous iron ion concentration increased, but when we added the Fer-1 to the OE_circ_BBS9 group, ferrous iron ion concentration decreased (**Figure 4G**). These results demonstrate that OE_circ_BBS9 promotes ferroptosis in LUAD cells.

Glutathione peroxidase 4 (GPX4) mitigates the toxicity of lipid peroxides through its catalytic activity, maintaining the stability of the lipid bilayer membrane, thereby inhibiting the occurrence of ferroptosis. Ferritin heavy chain 1 (FTH1) can disrupt autophagosomes, thus inhibiting ferroptosis. The expression of GPX4 and FTH1 was assessed through Western blot analysis. In H1299 cells, the introduction of the erastin led to decreased expression of GPX4 and FTH1, while adding the erastin inducer to the si_circ_BBS9 group resulted in increased expression of GPX4 and FTH1 (**Figure 4H**). These findings indicate that si_circ_BBS9 inhibits ferroptosis in LUAD cells. In A549 cells overexpressing OE_circ_BBS9, the expression of GPX4 and FTH1 decreased, but when we added the Fer-1 to the OE_circ_BBS9 group, the expression of GPX4 and FTH1 increased (**Figure 4I**). These results demonstrate that OE_circ_BBS9 promotes ferroptosis in LUAD cells.

These experimental results underscore the role of circ_BBS9 in regulating the process of ferroptosis and its impact on lipid oxidation and iron ion homeostasis. This contributes to a deeper understanding of the biological mechanisms of circ_BBS9 in LUAD.

### Circ_BBS9 interacts with IFIT3, mediating protein ubiquitination and subsequently affecting protein stability

**Figure 5:**
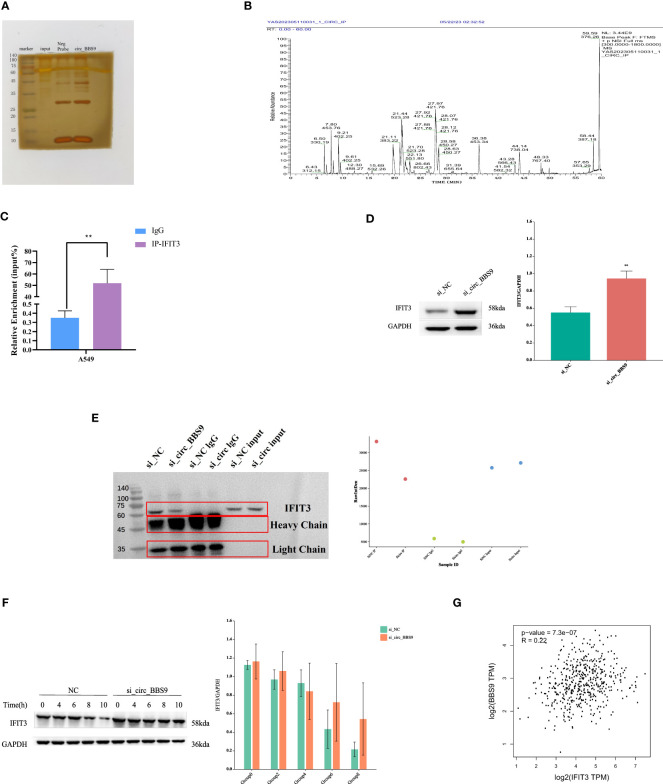
Circ_BBS9 interacts with IFIT3, mediating protein ubiquitination and subsequently affecting protein stability. **(A)** RNA pulldown was performed using biotin-labeled circ_BBS9 probe, followed by mass spectrometry analysis. **(B)** RNA pulldown assay was performed to verify the interaction between circ_BBS9 and IFIT3 in A549 cell. **(C)** RBP immunoprecipitation (RIP) assay was performed using IG-IFIT3 or IgG antibodies, followed by qRT-PCR assay for circ_BBS9 expression in A549 cell. **(D)** Western blot was performed to detect the expression of IFIT3 in GAPDH and si_circ_BBS9. **(E)** Co-immunoprecipitation (Co-IP) experiments were conducted on A549 cells using ubiquitination antibodies. **(F)** Experiments on the stability of IFIT3 protein. **(G)** Gene expression profiling interactive analysis (GEPIA: http://gepia.cancerpku.cn/).

To identify potential proteins that interact with circ_BBS9 in LUAD, we employed biotinylated circ_BBS9 probes for circ_RNA pull-down analysis, followed by silver staining SDS-PAGE analysis (**Figure 5A**). Bands of proteins that differed between the Control and circ_BBS9 groups were excised and sent to the mass spectrometry platform for protein identification (**Figure 5B**).

Among the potential proteins interacting with circ_BBS9, circ_RNA pull-down and immunoprecipitation (IP) cell lysate silver staining experiments revealed a successful pull-down of circ_BBS9. Furthermore, these experiments demonstrated a robust binding between circ_BBS9 and IFIT3 protein within A549 cells (**Figure 5A**). Additionally, RNA immunoprecipitation (RIP-PCR) experiments showed the enrichment of circ_BBS9 within the IP-IFIT3 group, providing further support for the molecular interaction between circ_BBS9 and IFIT3 (**Figure 5C**). Moreover, Western blot (WB) analysis of the pulled-down proteins revealed a significant increase in IFIT3 protein levels in A549 cells with circ_BBS9 knockdown (**Figure 5D**).

In addition, we performed co-immunoprecipitation experiments with A549 cells using a ubiquitination antibody (**Figure 5E**) and assessed IFIT3 protein stability (**Figure 5F**). Consequently, we postulate that circ_BBS9 may stabilize the IFIT3 protein through direct interaction.

To further validate our findings, we utilized Gene Expression Profiling Interactive Analysis (GEPIA) to confirm this association. Our analysis revealed a positive correlation between BBS9 and IFIT3 in LUAD (**Figure 5G**). These results provide experimental evidence for the interaction between circ_BBS9 and IFIT3, emphasizing its potential significance in LUAD.

### Construction of the upstream regulatory network of IFIT3

**Figure 6:**
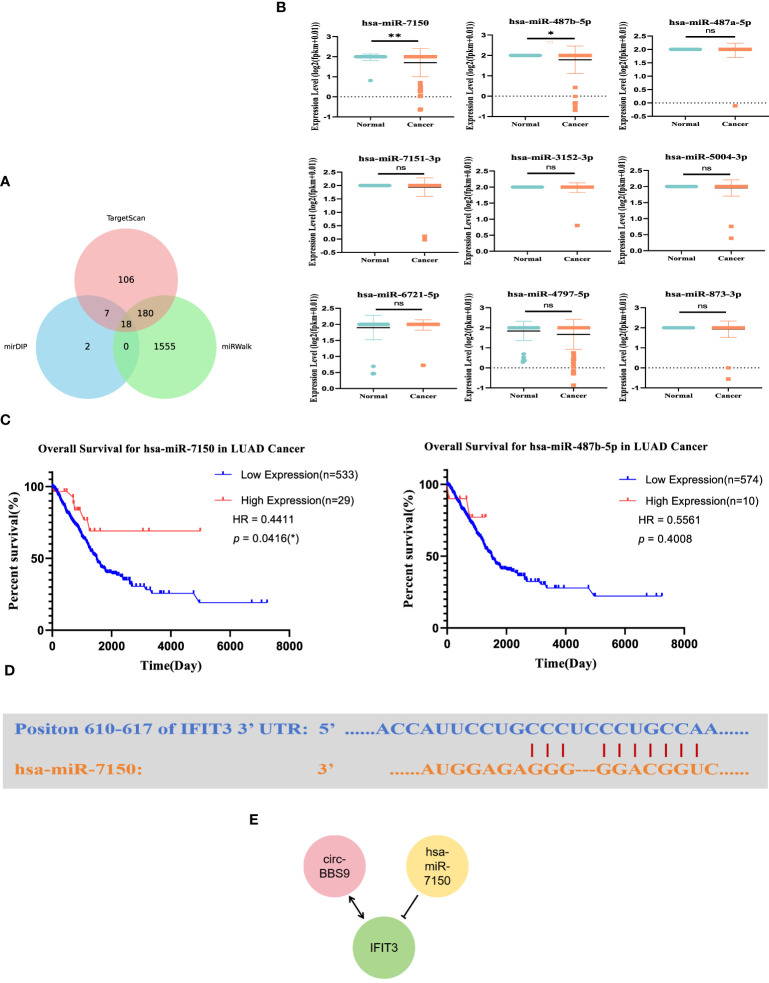
Construction of the Upstream Regulatory Network of IFIT3. **(A)** Venn diagram found that potential miRNAs of IFIT3. **(B)** Predicted miRNA expression levels. **(C)** Predicted miRNA survival levels. **(D)** Predicted interaction of IFIT3 and has-miR-7150. **(E)** Potential upstream TF-miRNA-mRNA regulatory network of IFIT3.

We utilized miRNA databases, mirDIP, and TargetScan resources to explore the upstream miRNA regulatory factors controlling IFIT3 (**Figure 6A**). Initially, through these databases, we obtained a set of predicted miRNAs, totaling 18. Subsequently, we conducted expression and survival analysis for these predicted miRNAs (**Figures 6B, C**) to further refine our selection. During the analysis, it became evident that only 9 of the miRNAs had available expression data. Therefore, we performed statistical analysis on the expression of these 9 miRNAs, with particular focus on hsa-miR-7150 and hsa-miR-487b-5p. We also conducted statistical analysis of their overall survival. The results indicated that hsa-miR-7150 was the most likely miRNA involved in the regulation of IFIT3.Furthermore, we provided the complementary sequences between IFIT3 and hsa-miR-7150 (**Figure 6D**), implying the potential existence of a miRNA-mRNA interaction between them.

Based on these findings, we constructed a molecular regulatory network in LUAD (**Figure 6E**), which includes circ_BBS9, hsa-miR-7150, and IFIT3. This network aids in gaining a better understanding of the regulatory mechanisms of IFIT3 and its role in LUAD.

### The relationship between IFIT3 expression and the prognosis of LUAD

**Figure 7:**
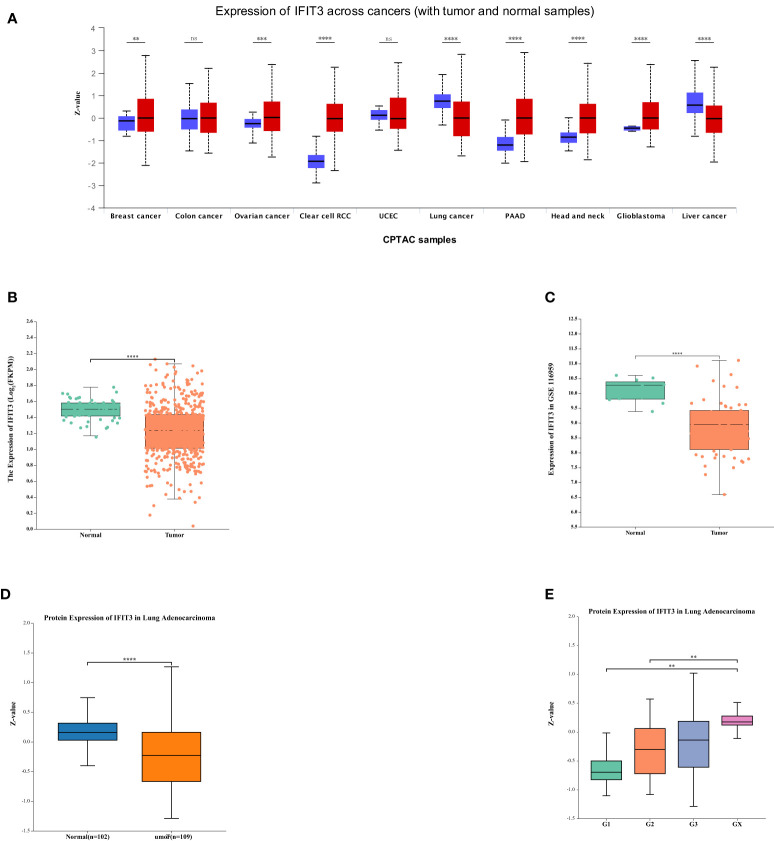
The Relationship Between IFIT3 Expression and the Prognosis of LUAD. **(A)** IFIT3 expression in different cancer types analyzed by UALCAN. **(B)** IFIT3 mRNA is expressed at a low level in TCGA. **(C)** IFIT3mRNA is expressed at a low level in GEO. **(D)** In the HAP database, the protein expression level of IFIT3 in LUAD is significantly lower than in normal tissues (P < 0.001). **(E)** The expression of IFIT3 in LUAD is significantly correlated with Tumor Grade (*p* < 0.01).

To establish the relationship between IFIT3 and LUAD, we conducted a series of data analyses. First, we used UALCAN to analyze the expression of IFIT3 in different tumors (**Figure 7A**). The results showed differential expression of IFIT3 in various tumor tissues. We analyzed data from the TCGA database (**Figure 7B**) and the GSE116959 GEO database (**Figure 7C**), assessing the mRNA expression levels of IFIT3 in LUAD tissue and non-tumor tissue, and validated the protein-level expression of IFIT3 in LUAD using the Human Protein Atlas (HPA) database (**Figure 7D**).

Through the analysis from these different databases and methods, we consistently observed significant downregulation of IFIT3 in LUAD tissues, indicating that IFIT3 expression in LUAD is negatively regulated. Furthermore, we found that the expression levels of IFIT3 increased with higher tumor grades (**Figure 7E**), suggesting a correlation between IFIT3 and tumor grade, indicating its potential role in the development of LUAD.

These analytical results strengthen the association between the downregulation of IFIT3 expression and tumor progression in LUAD, providing important clues for further research into the function and potential role of IFIT3.

### GO and KEGG analysis related to IFIT3

**Figure 8:**
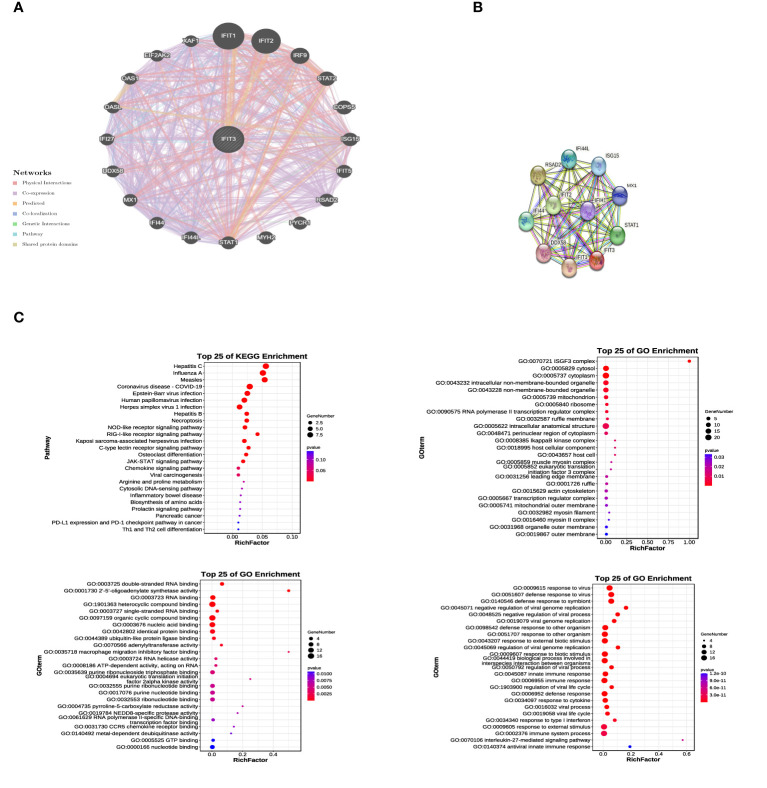
GO and KEGG Analysis Related to IFIT3. **(A)** The gene network of GPER and associated genes was analyzed using Gene Mania. **(B)** The protein network of GPER was constructed using STRING. **(C)** 21 related genes were used for conducting GO and KEGG analysis.

Through the Gene-Gene interaction network in GeneMania (**Figure 8A**) and the construction of a Protein-Protein Interaction (PPI) network using the STRING database (**Figure 8B**), we identified 20 genes associated with IFIT3. These 21 related genes were utilized for performing GO and KEGG analysis (**Figure 8C**) to gain a deeper understanding of the biological functions and pathways associated with IFIT3.

In the analysis results, the top 25 KEGG pathways, cellular components (CC), biological processes (BP), and molecular functions (MF) were identified. These pathways include the JAK-STAT signaling pathway and cytokine signaling in the immune system. These findings provide valuable insights into the significant roles of IFIT3 and its related genes in cellular signal transduction and immune system functions. They contribute to a better understanding of their functions and regulatory mechanisms. These bioinformatics analyses offer a strong basis for further experimental research.

### The research on the correlation between the expression of IFIT3 and immune infiltration

**Figure 9:**
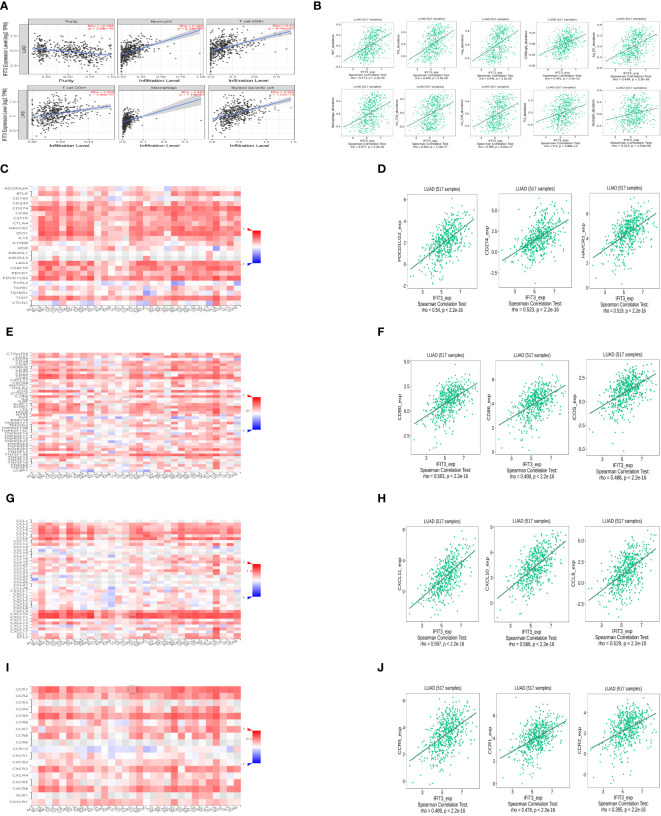
The research on the correlation between the expression of IFIT3 and immune infiltration. **(A)** Relationship between IFIT3 expression and immune infiltration level generated from TIMER. **(B)** Correlation of IFIT3 expression and immune infiltration from TISIDB. **(C)** Correlations between IFIT3 expression and immune inhibitors in all kinds of human cancers analyzed by TISIDB. **(D)** The IFIT3 expression was positively associated with most of the immuneinhibitors. **(E)** Correlations between IFIT3 expression and immuneostimulators in all types of human cancers analyzed by TISIDB. **(F)** The IFIT3 expression was positively associated with most of the immunostimulators. **(G)** Correlations between IFIT3 expression and chemokines in all types of human cancers analyzed by TISIDB. **(H)** The IFIT3 expression was positively associated with most of the chemokines. **(I)** Correlations between IFIT3 expression and chemokine receptors. in all types of human cancers analyzed by TISIDB. **(J)** The IFIT3 expression was positively associated with most of the chemokine receptors.

The preliminary investigation of IFIT3’s involvement in immune infiltration was carried out using the TIMER database (**Figure 9A**). The results indicate that IFIT3 positively correlates with various immune cells, including neutrophils, CD8+ T cells, macrophages, dendritic cells, CD4+ T cells, suggesting that IFIT3 regulates the infiltration of different immune cells. Spearman correlation and p-values were used for this analysis. Further exploration of the correlation between IFIT3 expression and immune infiltration was conducted using the TISIDB (**Figure 9B**), showing positive correlations with various immune cells, including NKT cells, Th1 cells, Treg cells, CD56 cells, activated dendritic cells, macrophages, activated CD4 T cells, activated CD8 T cells, Th2 cells, and neutrophils. The expression of IFIT3 is associated with the regulation of immune infiltration-related immune factors, immune subtypes, and immune cells.

Additionally, the analysis was extended to investigate the correlation between IFIT3 expression and immune modulators (**Figure 9C**), immune stimulators (**Figure 9E**), chemokines (**Figure 9G**), and chemokine receptors (**Figure 9I**). The results revealed a positive correlation between IFIT3 expression and most immune modulators (**Figure 9D**). In LUAD, the top three positive correlations were observed with PDCD1LG2 (rho = 0.54, *P* < 2.2e-16), CD274 (rho = 0.523, *P* < 2.2e-16), and HAVCR2 (rho=0.519, *P*<2.2e-16). Similarly, a positive correlation was found between IFIT3 expression and most immune stimulators (**Figure 9F**), with the top three positive correlations in LUAD being CD80 (rho = 0.503, *P* < 2.2e-16), CD86 (rho = 0.498, *P* < 2.2e-16), and ICOS (rho=0.488, *P*<2.2e-16). Furthermore, a positive correlation was identified between IFIT3 expression and most chemokines (**Figure 9H**), with the top three positive correlations in LUAD being CXCL11 (rho=0.597, *P*<2.2-16), CXCL10 (rho=0.566, *P*<2.2-16), and CCL8 (rho=0.529, *P*<2.2-16). Finally, a positive correlation was observed between IFIT3 expression and most chemokine receptors (**Figure 9J**), with the top three positive correlations in LUAD being CCR5 (rho = 0.489, *P* < 2.2e-16), CCR1 (rho = 0.476, *P* < 2.2e-16), and CCR2 (rho=0.395, *P*<2.2e-16).

## Discussion

Research on Circular RNA (circRNA) represents an emerging field that has garnered significant attention owing to rapid technological progress. CircRNAs exert pivotal roles in a diverse range of physiological and pathological processes. Their critical implications in cancer initiation, progression, and the development of drug resistance have been well-documented (42, 43). Furthermore, their prevalence in exosomes and bodily fluids enables them to modulate the tumor microenvironment via intercellular communication. As a result, strategies based on circRNA for diagnosis and therapeutics carry immense potential in cancer management and are poised to emerge as highly promising cancer biomarkers.

The dysregulated expression of circular RNAs (circRNAs) exerts a substantial impact on tumor development. In this study, we integrated data from GEO databases, conducted microarray analyses, and scrutinized clinical samples to identify the most significantly downregulated circRNA candidate genes. Following meticulous data screening, we recognized the importance of constructing a comprehensive framework to delineate the involvement of circ_BBS9 in the malignant progression and immune regulation of LUAD. Our research extensively delved into the role of circ_BBS9 in LUAD, examining aspects such as gene expression patterns, molecular functions, and immune infiltration within the context of the disease.

The results from bioinformatics analyses conducted across various databases and utilizing different methods consistently revealed a notable downregulation trend in circ_BBS9 expression within LUAD cells and tissues (**Figures 1B, C**, **2F, G**). Subsequent validation using clinical samples substantiated these findings, demonstrating significantly lower circ_BBS9 expression levels in LUAD tissues in comparison to non-tumor tissues (**Figures 2A, B, D**). Furthermore, the observed decrease in circ_BBS9 expression corresponded with higher tumor grades (**Figure 2H**), implying a potential correlation between reduced circ_BBS9 expression levels and an unfavorable prognosis in LUAD (**Figures 2I, J**). Consequently, our findings suggest a plausible association between the loss of circ_BBS9 and the progression of LUAD. Annotation results from GO and KEGG analysis further indicate that circ_BBS9 is involved in the regulation of cellular metabolism, biological regulation, cytokine interactions, and various signaling pathways (**Figures 1D, E**). GSEA results also show a close association between circ_BBS9 and various pathways including cellular metabolism, the cell cycle, proliferation, infiltration, antigen response, inflammatory reactions, and multiple signaling pathways, such as the P53, WNT, NF-кB signaling pathways, and the G2M checkpoint (**Figure 1F**). Our study demonstrates that the expression of circ_BBS9 is correlated with patient prognosis and tumor grade. Additionally, our laboratory experiments confirm that the overexpression of circ_BBS9 can significantly inhibit the proliferation of LUAD cells (**Figures 3E, F**), suggesting its potential as a tumor suppressor in LUAD.

CircRNAs have been associated with drug resistance in hepatocellular carcinoma (HCC) cells and their regulatory roles in autophagy (44, 45). Previous research has suggested that circRNAs may play a vital role in autophagic regulation, prompting further exploration of their potential roles in ferroptosis. Recent studies have confirmed that circular RNAs can promote or inhibit ferroptosis by modulating the post-transcriptional levels of key ferroptosis-related proteins, through different molecular pathways, which contribute to the progression of various cancers. The molecular mechanism of ferroptosis primarily involves the loss or activation of GPX4, iron metabolism, and lipid peroxidation. One study discovered that in lung cancer, the overexpression of circ_SCN8A led to elevated levels of intracellular Fe2+, ROS, and MDA while decreasing the levels of glutathione (GSH). Additionally, circ_SCN8A enhances the expression of fatty acid-binding protein 4 (ACSL4) by binding to miR-1290, promoting lung cancer cell proliferation and migration, and inhibiting ferroptosis (46). Hence, we believe that ferroptosis regulated by circRNAs could become a novel cancer treatment strategy. However, our understanding of the mechanisms of circRNAs in ferroptosis regulation remains incomplete. In our study, overexpression of circ_BBS9 experimentally validated significantly increased ROS levels (**Figure 4C**), elevated MDA and divalent iron ion concentrations (**Figures 4E, G**), and reduced the expression levels of GPX4 and FTH1 (**Figure 4I**), further confirming the role of circ_BBS9 in promoting ferroptosis in LUAD cells. These findings suggest that circ_BBS9 holds potential as a biomarker and therapeutic target for LUAD, offering a new avenue for research into ferroptosis-induced treatments for LUAD. Currently, some drugs and radiotherapy have been found to induce cell ferroptosis, making RNA molecules an area of significant interest in research. Our study provides a new research direction for exploring the potential of circ_BBS9 as a biomarker and molecular therapy for LUAD.

Ferroptosis has become a significant focus in contemporary cancer research. While directly manipulating ferroptosis pathways may not be the most effective overall strategy, revealing its regulatory pathways establishes a new theoretical foundation for precise and targeted cancer therapies. The revelation of ferroptosis has inaugurated novel research trajectories within the cancer domain, gradually unveiling its clinical significance in cancer progression. Hence, intervening in cancer progression by modulating cellular ferroptosis has become a pivotal area of investigation in research. It is crucial to emphasize that our study remains in its nascent stages, particularly regarding the exploration of mechanisms through which circ_BBS9 regulates ferroptosis in LUAD. Consequently, further exploration, including validation in more extensive cohorts of lung cancer patients and diverse lung cancer tissue samples, alongside rectifying systematic biases inherent in various databases, constitutes an integral part of our future research endeavors.

In recent years, the advent and efficacy of targeted immunotherapies have begun to reshape the management of cancer (47, 48). Infiltrative immune cells are a crucial component of the TME (49). The interaction between the TME and the host immune system is complex, necessitating the identification of predictive biomarkers for personalized treatment. Infiltration of innate immune cells and the production and aggregation of inflammatory chemokines are often indicative of tumor-associated inflammation. Immune-inflammatory responses can activate a cascade of molecular signaling pathways associated with tumor cell generation, proliferation, and metastasis (50–52). Furthermore, distinct subtypes of the tumor microenvironment (TME) are significantly correlated with patient prognosis, predicting the immune response rate and sensitivity to chemotherapy drugs. This information can be utilized to screen patients for sensitivity to immunotherapy and chemotherapy drugs (53). It is widely recognized that an imbalance in T-cell subpopulations is common in cancer patients (54). The cytokines and chemokines secreted by TH1 cells play a major role as effector molecules in immune cell signaling. Some studies have found that TH1 cells can promote anti-tumor immune responses, reduce cancer cell proliferation, and guide them into a dormant state by activating the STAT1 signaling pathway (55).

With the deepening investigation into circRNAs, an increasing body of research has shown that circRNAs can interact with RNA-binding proteins (RBPs) to modulate cancer progression. For instance, circ_NDUFB2 has been demonstrated to impact the occurrence and development of non-small cell lung cancer by regulating the ubiquitination and degradation of IGF2BP2 (14). In this study, we conducted microarray analysis of the expression profile of circRNAs using a LUAD expression chip dataset. Through circRNA pull-down and mass spectrometry detection, we successfully identified the protein IFIT3 (**Figures 5A–C**), which directly interacts with circ_BBS9. We observed that IFIT3 is under-expressed in LUAD (**Figures 7B–D**) and increases with tumor grade (**Figure 7E**). Additionally, IFIT3 exhibits differential expression across various tumor tissues (**Figure 7A**). Through GO and KEGG analysis of 21 genes related to IFIT3 (**Figures 8A, B**), it is shown (**Figure 8C**) that IFIT3 plays a crucial role in cytokine signaling pathway in the JAK-STAT signaling pathway and immune system. Moreover, we explored the upstream miRNA regulatory factors for IFIT3 using tools like miRNA databases, mirDIP, and TargetScan (**Figures 6A–D**). From these investigations and considering expression and survival data from databases, we have identified a potential transcriptional network involving “circ_BBS9,” “hsa-miR-7150,” and “IFIT3,” suggesting its potential involvement in the pathogenesis of LUAD. It may exert its effects by modulating ferroptosis and immune microenvironment through direct interaction with IFIT3 and competitive binding to miR-7150. A study has revealed the upregulation of hsa-miR-7150 expression in tissue samples from advanced gastric cancer patients (56).

It’s worth noting that this transcriptional network was selected through bioinformatics analysis and preliminary functional exploration, and further refinement of this network will be required in future studies. Establishing the functional transcriptional network of LUAD requires the utilization of new effective methods and bioinformatics tools, as well as more *in vitro* and *in vivo* experiments to reveal its potential pathogenic mechanisms and identify new diagnostic biomarkers. Therefore, future research may involve further experimental investigations to delve into the associated molecular mechanisms.

Following the construction of this network, we conducted further analysis with the aim of investigating the association between IFIT3 in LUAD and immune infiltration to explore the link between gene expression and immune cell infiltration. Although prior research has indicated the potential impact of immune infiltration on the behavior and prognosis of cancer patients, the mechanism of interaction between IFIT3 and the TME remains unclear. Our analysis reveals that the expression of IFIT3, a protein that directly interacts with circ_BBS9, is positively correlated with the infiltration of T-helper cells and Th1 cells (**Figure 9B**). Furthermore, in the IFIT3-related PPI network, STAT1, recognized as a critical downstream factor of Th1 cells, is identified. IFIT3 also exhibits differential expression across various tumors. Previous studies have suggested that IFIT3 enhances the interferon (IFN) effector signaling pathway by promoting the formation and nuclear localization of the STAT1-STAT2 heterodimer in hepatocellular carcinoma (57). Our analysis also indicates a positive correlation between IFIT3 expression and immunosuppressive cells, like Treg cells (**Figure 9B**), and immunosuppressive molecule CD274 (**Figure 9D**). Meanwhile, immune-activated cells and markers, such as anti-tumor T cells, neutrophils, and Th1 cells, also exhibit a positive correlation with IFIT3 expression (**Figure 9A**). All of these findings support the potential of IFIT3 as a marker or target for distinguishing malignant tumors. Therefore, the regulation of IFIT3 expression at the genetic level may provide a new target for immunotherapy in LUAD.

Current research suggests that CD80 is a potential therapeutic target for improving the prognosis of patients with LUAD and enhancing the effectiveness of biologically targeted anti-tumor treatments. CXCR3, a receptor for the chemokine CXCL11, demonstrates strong anti-tumor activity *in vivo* (58). Blocking CCR5 to promote the polarization of anti-tumor macrophages has been observed to lead to the regression of metastatic disease and alterations in the TME (59). Our study results indicate that IFIT3 is positively correlated with certain immune molecules such as PDCD1LG2, CD80, CD86, CXCL11, CXCL10, and CCR5 (**Figures 9F, H, J**). Among these, CXCL11, CXCL10, and CCR5 are chemokines and related receptors for Th1 cells. These findings support the hypothesis that IFIT3 may serve as a potential marker or therapeutic target for malignant tumors.

To delve deeper into the regulatory mechanisms of IFIT3 in malignant tumor progression and immune infiltration, we combined the KEGG results and identified a potential pathway: the JAK3-STAT pathway, which may be associated with IFIT3 (**Figure 8C**). Activation of the JAK-STAT signaling pathway could suppress cytotoxic T lymphocytes and counteract the anti-tumor effects of PD-1 immune therapy in pancreatic cancer (60). Inhibition of LUAD proliferation, migration, and invasion can be achieved by lowering PYCR1 expression, affecting the JAK/STAT signaling pathway (61). Several studies have reported that the activation of the JAK-STAT signaling pathway can promote cell apoptosis (62, 63). However, the JAK-STAT signaling pathway plays a dual role in the TME, acting as both “anti-tumor” and “pro-tumor” depending on the nature of the response signals. Therefore, we hypothesize that IFIT3 may exert its effects through the JAK-STAT signaling pathway and show a strong positive correlation with immune molecules, thereby playing a role in the regulation of progression and immune infiltration in LUAD.

We comprehensively elucidated the role of IFIT3 in pathway enrichment and immune infiltration within the TME using bioinformatics techniques. Its low expression may serve as an indicator of poor prognosis in LUAD and potentially enhance the effectiveness of immune therapy through the regulation of immune cell infiltration. Therefore, the decreased expression of circ_BBS9 and IFIT3 in LUAD may impact tumor immunity and contribute to tumorigenesis, providing important insights for future immunotherapy research. This also suggests that disease treatment should not only focus on the molecular level but should also be combined with an emphasis on immune infiltration to achieve better therapeutic outcomes. In summary, our data indicates a strong association between IFIT3 and various immune checkpoint molecules and immune activation within the TME. Nevertheless, further validation of IFIT3 as a predictive biomarker for selecting immune checkpoint blockade and the potential utility in treating patients with immunotherapy is required. Consequently, large-scale standardized animal experiments, clinical trials, and additional immunotherapy cohorts and single-cell analyses are necessary, and our team is actively working towards these objectives.

The development and progression of LUAD involve a multifaceted, multistep process primarily steered by aberrant gene expression within cellular signaling pathways. Constructing a high-dimensional immune map that integrates complementary predictive biomarkers becomes paramount for personalized immune therapy, given the intricate interplays among tumors, the tumor microenvironment (TME), and the host’s immune responses. Hence, collaborative efforts in the future are imperative to ensure the efficacy of immune therapeutic approaches targeted toward appropriate TMEs at specific intervention junctures. Our study delved into immune-related genes implicated in formulating the LUAD prognosis model, comprehensively analyzing associated immune cells and immune signaling pathways. Despite the comprehensiveness of our research, these conclusions await full validation through *in vitro* or *in vivo* experiments due to inherent limitations. Therefore, further research endeavors are essential to elucidate the precise functions of these pathways, enhancing and confirming the stability of these regulatory networks to solidify their mechanistic underpinnings. We identified that the upregulation of circ_BBS9 impedes the proliferation of lung adenocarcinoma cells and encourages ferroptosis in these cells. Additionally, we established a relationship between the protein IFIT3, which directly interacts with circ_BBS9, and immune infiltration, contributing to the configuration of the immune microenvironment in LUAD. Furthermore, we underscored the potential of circ_BBS9 as a novel biomarker for early diagnosis and treatment, unveiling the direct interplay between circ_BBS9 and IFIT3, which actively shapes the immune microenvironment in LUAD. These investigations provide novel insights into molecular mechanisms and prospective therapeutic targets, holding substantial promise in the diagnosis and treatment landscape of LUAD.

## Conclusion

Circ_BBS9 acts as a tumor suppressor in LUAD and may serve as a potential diagnostic biomarker. It may exert its effects by modulating ferroptosis and immune microenvironment through direct interaction with IFIT3 and competitive binding to miR-7150. These findings provide novel insights into LUAD pathogenesis and identify circBBS9 as a promising therapeutic target.

## Data availability statement

The datasets presented in this study can be found in online repositories. The names of the repository/repositories and accession number(s) can be found in the article/supplementary material.

## Ethics statement

This study was conducted in accordance with the principles outlined in the Declaration of Helsinki and was approved by the Ethics Committee of Jinshan Branch of Shanghai Sixth Peoples Hospital, with approval number jszxyy202205. All patients provided written informed consent prior to their participation in this study. The study adhered to the ethical guidelines and regulations governing research involving human subjects and protected their rights and privacy throughout the investigation. All patient data and information were anonymized and treated with strict confidentiality to ensure compliance with ethical standards.

## Author contributions

DP: Conceptualization, Formal analysis, Funding acquisition, Investigation, Methodology, Project administration, Resources, Validation, Writing – original draft, Writing – review & editing. ML: Data curation, Formal analysis, Methodology, Software, Validation, Visualization, Writing – original draft, Writing – review & editing. LL: Investigation, Writing – review & editing. HY: Investigation, Writing – review & editing. DF: Investigation, Writing – review & editing. LC: Investigation, Writing – review & editing. BG: Conceptualization, Formal analysis, Funding acquisition, Investigation, Resources, Supervision, Writing – review & editing.
